# Optimal timing of ileostomy closure after rectal surgery: A Bayesian network meta-analysis of randomized controlled trials

**DOI:** 10.1007/s00423-026-04133-z

**Published:** 2026-07-09

**Authors:** Zikai Zhou, Siyi Wang, Jiahao Zhou, Jun Huang, Xiangbing Deng, Ziqiang Wang, Wenjian Meng, Qingbin Wu

**Affiliations:** 1 Colorectal Cancer Center, Department of General Surgery, West China Hospital, Sichuan University, No. 37, Guo Xue Xiang, Chengdu, 610041 China; 2 West China School of Medicine, Sichuan University, Chengdu, 610041 China

**Keywords:** Ileostomy closure, Postoperative complications, Rectal cancer, Stoma-related complications, Wound complications

## Abstract

**Purpose:**

Temporary ileostomy is commonly performed to protect rectal anastomosis after rectal surgery, yet the optimal timing of reversal remains unsettled. This study compared postoperative outcomes among early, intermediate, and late ileostomy closure and assessed the safety of early closure in selected patients.

**Methods:**

PubMed, Web of Science, and Embase were systematically searched from inception to June 21, 2026. Randomized controlled trials comparing different timing strategies for ileostomy closure were included. Closure timing was categorized according to the reported median or mean interval from primary surgery to reversal as early (≤ 35 days), intermediate (> 35 and ≤ 120 days), and late (> 120 days) based on the median or mean interval. The primary outcomes were total postoperative complications and major complications. A Bayesian network meta-analysis was performed.

**Results:**

Ten randomized trials involving 871 patients (316 early, 312 intermediate, and 243 late) were included. Total postoperative complications were comparable among the three groups. Compared with intermediate closure, early closure was associated with higher risks of major complications (odds ratio [OR] 5.30, 95% credible interval [CrI] 1.30–63.00) and wound complications (OR 4.80, 95%CrI 1.10–35.00), but a lower risk of stoma-related complications (OR 0.086, 95%CrI 0.0057-0.82). No significant differences were observed between early and late closure or between intermediate and late closure. In patients without clinical or radiological evidence of anastomotic leakage, total and major complication rates were similar across groups.

**Conclusion:**

Early ileostomy closure may be feasible and safe in carefully selected patients without evidence of anastomotic leakage, reducing stoma-related complications at the expense of increased wound complications.

**Supplementary Information:**

The online version contains supplementary material available at https://doi.org/10.1007/s00423-026-04133-z.

## Introduction

Anastomotic leak remains one of the most consequential complication after rectal surgery, occurring in approximately 5–12% of patients and contributing substantially to postoperative morbidity, reintervention, and mortality [1–4]. The rationale for temporary faecal diversion is to reduce faecal contamination of the distal anastomosis and mitigate the clinical consequences of leakage. Although whether diversion lowers the absolute incidence of anastomotic leak remains debated [1, 5, 6], evidence more consistently suggests that it attenuates leak severity and reduces the need for reoperation once leakage occurs [7–9]. Accordingly, surgeons commonly fashion a defunctioning stoma in patients considered at increased risk for clinically consequential leakage, including those with obesity, low rectal cancer, or exposure to neoadjuvant therapy [2, 10, 11]. When diversion is required, loop ileostomy is often selected over colostomy because it is technically straightforward to construct and reverse and has been associated with fewer postoperative and stoma-related complications and faster bowel recovery [12].

Once anastomotic integrity has been confirmed and the patient is clinically fit, temporary ileostomy closure is generally considered. In contemporary practice, reversal is commonly scheduled approximately 8–12 weeks, or about 3 months, after the index operation, although no universally accepted timing threshold exists. Prolonged diversion, however, exposes patients to cumulative stoma-related morbidity. Reported complication rates vary widely, from approximately 3–60%, reflecting differences in patient selection, diversion duration, outcome definitions, and follow-up intervals; common events include stomal prolapse, stenosis, parastomal hernia, high-output stoma, and chronic renal injury [13–15]. Beyond local stoma morbidity, prolonged diversion can impair quality of life (QoL), and longer stoma duration has been associated with a higher risk of major low anterior resection syndrome (LARS), suggesting that reversal timing may have functional as well as perioperative implications [16–18].

To reduce the cumulative burden of living with a stoma, earlier closure has attracted increasing interest; however, its safety remains unsettled. Several randomized controlled trials (RCTs) have produced discordant findings. Some reported early closure to be feasible and safe [19, 20], whereas others were terminated prematurely due to excess morbidity [21–23]. One plausible explanation is that early closure has not been defined consistently across trials. Across the RCTs, the mean or median interval between primary surgery and ileostomy closure ranges from 8 to 250 days, generating clinically heterogeneous timing categories. This heterogeneity may partly explain why previous pairwise meta-analyses have reached inconsistent conclusions, with some reporting favorable results for early closure [24, 25], while others not confirming such benefit [26, 27]. Similarly, the recent meta-analysis by Emile et al. retained a binary comparison between early and standard closure and reported a higher risk of anastomotic leak after early closure, particularly when reversal occurred within 2 weeks [28].

What remains underexplored is whether intermediate closure and genuinely late closure should be treated as interchangeable components of a single delayed-closure comparator. Challenging the conventional binary framework of early versus delayed closure, we considered that an intermediate interval may represent a distinct clinical strategy rather than a residual category within delayed reversal. Bayesian network meta-analysis provides a methodologically appropriate framework for this question because it permits simultaneous comparison of multiple competing timing strategies by integrating direct and indirect randomized evidence [29–31]. The present investigation therefore offers a novel contribution by separating ileostomy closure into early, intermediate, and late strategies and comparing these clinically relevant intervals within a single Bayesian framework. We conducted a Bayesian network meta-analysis to compare early, intermediate, and late closure for ileostomy with respect to the postoperative complications.

## Methods

This Bayesian network meta-analysis was conducted and reported in accordance with PRISMA (Preferred Reporting Items for Systematic Reviews and Meta-Analyses) and AMSTAR (Assessing the methodological quality of systematic reviews) Guidelines [32, 33]. The protocol was established prospectively and registered in the Prospective Register of Systematic Reviews. Challenging the conventional binary classification of early versus delayed ileostomy closure, we categorized closure timing into three predefined intervals according to the median or mean time from primary rectal surgery to ileostomy closure: early (≤ 35 days), intermediate (> 35 and ≤ 120 days), and late (> 120 days) groups.

### Study selection

A systematic literature search was performed using the PubMed, Web of Science, and Embase databases. The search strategy is summarized in Table S1. The search was performed on November 15, 2023 and was updated on June 21, 2026. To minimize retrieval bias, we also manually screened the reference lists of eligible studies and relevant reviews for additional randomized trials .

### Inclusion and exclusion criteria

In this network meta-analysis, we only included RCTs that compared different time intervals between rectal surgery and ileostomy closure (early, intermediate, and late closure). Studies were included if they met the following criteria: (1) participants were patients undergoing rectal surgery with creation of a temporary diverting ileostomy; (2) the intervention and comparator were different timing strategies for ileostomy closure, categorized as early, intermediate, or late closure according to the predefined time intervals; (3) relevant postoperative outcome was reported, including total complications, major complications, anastomotic leak, wound complications, stoma-related complications, reoperation, readmission, operative time, length of hospital stay, bowel function, or quality of life. Studies were excluded if they were non-randomized studies, reviews, meta-analyses, conference abstracts, comments, letters, editorials, protocols without extractable outcome data, animal studies, or duplicate publications without additional usable data. Studies involving defunctioning colostomy rather than ileostomy, or those not comparing different ileostomy closure timing strategies, were also excluded. More detailed inclusion and exclusion criteria based on the PICO framework are provided in the Supplementary Methods.

### Data extraction

Data on study characteristics and outcomes were extracted from the included RCTs, as described in Supplementary Methods. The primary outcomes were total postoperative complications and major postoperative complications. Major complications were defined as Clavien-Dindo grade IIIa or higher. Secondary outcomes are described in the Supplementary Methods.

### Quality assessment

The standard Jadad scoring system and the Cochrane Risk Assessment Tool were used to assess the quality of the enrolled RCTs (Supplementary Methods) [34, 35].

### Data synthesis and analysis

A direct meta-analysis with a random-effects model was conducted to calculate the odds ratios (OR) and weighted mean differences (WMD) with corresponding 95% confidence intervals (95%CI) for each pairwise comparison between the early, intermediate, and late groups. A random-effects Bayesian network meta-analysis using Markov chain Monte Carlo methods and uninformative priors was conducted for each outcome [36, 37]. A network plot was drawn to show direct and indirect comparisons among the three groups. Four chains were selected to fit the model with 100,000 simulation iterations after a burn-in of 50,000 iterations and a thinning interval of 10 for each chain to achieve convergence of the model. The convergence and trajectory density diagrams were constructed to evaluate the convergence and stability of the models, respectively. The node-splitting method was used to evaluate the network consistency by comparing direct and indirect estimates [38]. Direct and indirect comparisons was combined to estimate the outcome, reported as OR/WMD with 95% credible interval (CrI). The surface under the cumulative ranking curve (SUCRA) was calculated to show the rank probability of the three groups, which ranged from 0 (worst) to 1 (best) [39]. The Grading of Recommendations Assessment, Development and Evaluation (GRADE) criteria for network meta-analysis was used to evaluate the quality of evidence [40]. We conducted several subgroup analyses to evaluate the robustness and reliability of the results. The R software (version 3.6.2, Foundation for Statistical Computing, Vienna, Austria) with the gemtc package (version 1.0–1) was used for all Bayesian network meta-analysis [41, 42]. The detailed data synthesis and analysis are described in Supplementary Methods.

## Results

### Study selection

**Fig. 1:**
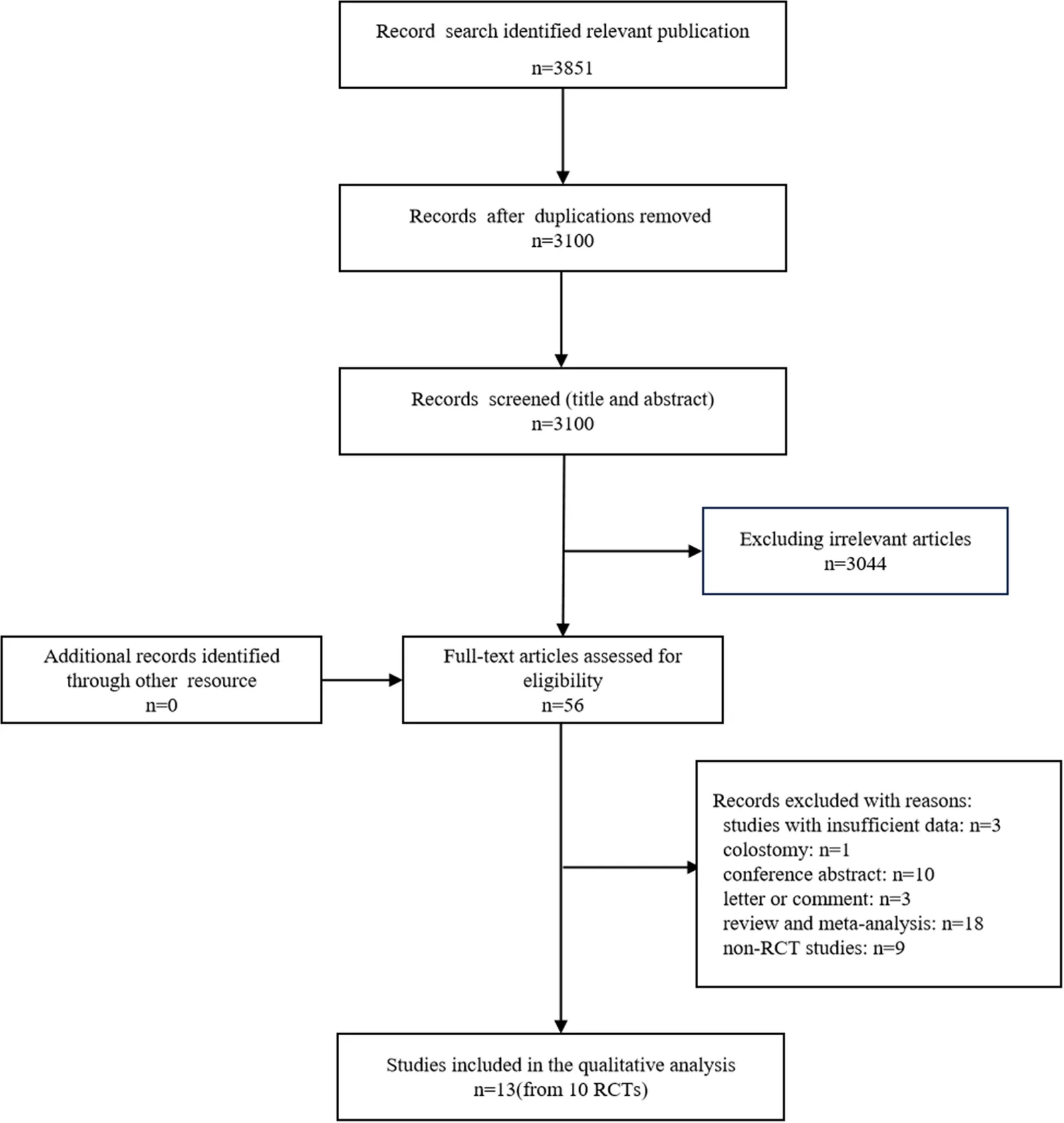
PRISMA flow chart showing selection of articles for review

The study selection process is shown in Fig. 1 and described in the Supplementary Results. Thirteen publications representing ten RCTs were included in our network meta-analysis. Of these, six articles from 5 RCTs compared early with intermediate closure [20–23, 43, 44], five articles from 3 RCTs compared early with late closure [17, 19, 45–47], and two articles from 2 RCTs compared intermediate with late closure [48, 49].

### Study characteristics and quality assessment

**Fig. 2:**
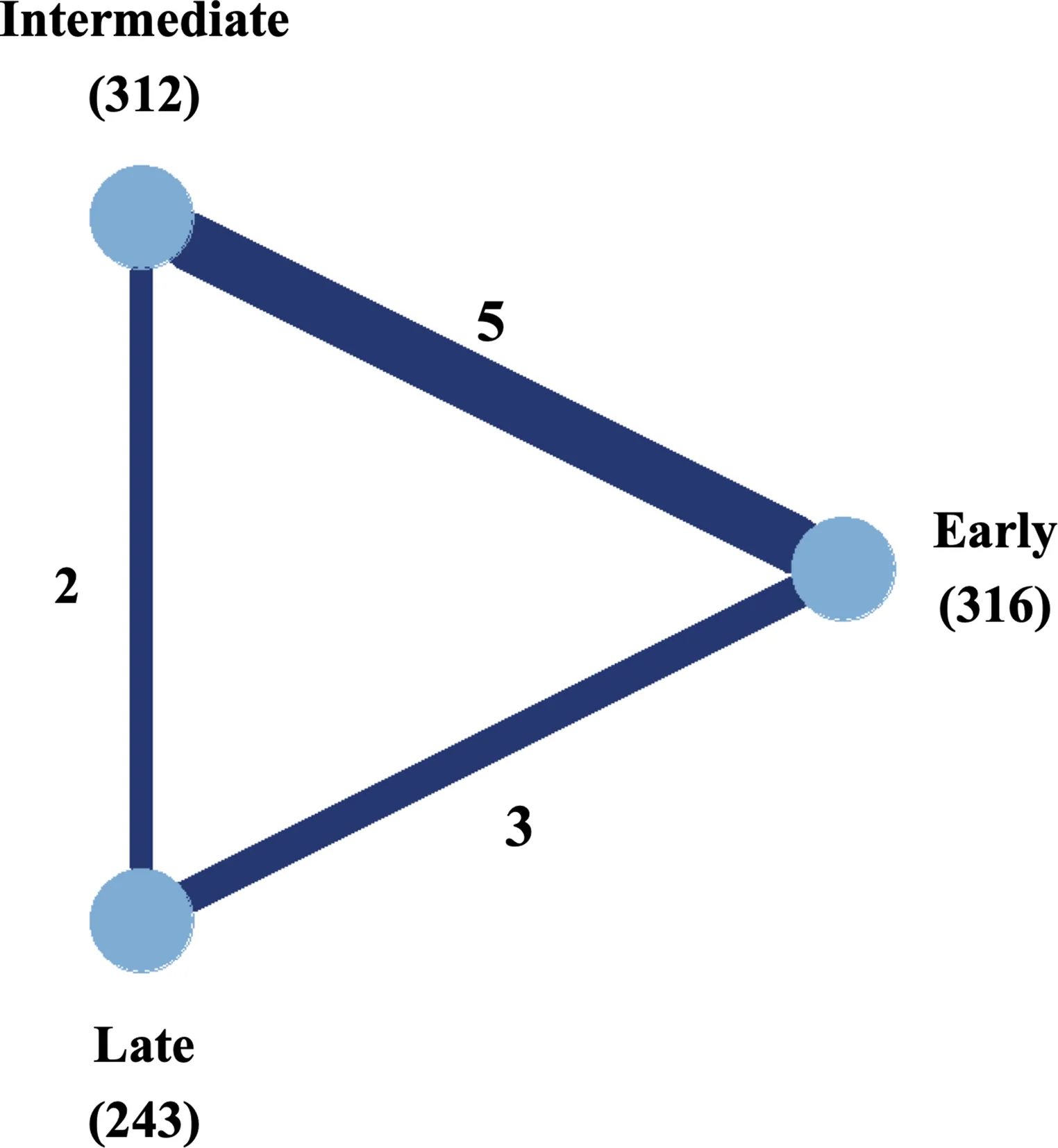
Network comparisons for the Bayesian network meta-analysis

Baseline characteristics are summarized in Table 1, Supplementary Results and Table S2. A total of 871 participants were included in the analysis: 316 in the early group, 312 in the intermediate group, and 243 in the late group. The network plot for the total postoperative complications is shown in Fig. 2. Network plots were constructed for all the other outcomes. There was no evidence of inconsistency in any of the network analyses, because the difference in DIC values between the consistency and inconsistency models was not significant. A quality assessment of the included RCTs is reported in the Supplementary Results.

**Table 1:** Baseline characteristics of included studies

Study	Country	Study period	Time points of randomization	Participation center	Primary bowel disease	Time intervals (days)	Groups	Sample size	Age (years)	Male gender	Outcomes
Alves 2008	France	2001–2004	After evaluating the anastomosis	Multicenter	Rectal cancer, IBD, FAP, and endometriosis	8(8–10) ^#^	Early	95	58(18–89) ^#^	44	1, 2, 3, 4, 5, 6, 8, 10, 11, 12, 14
						66(62–69) ^#^	Intermediate	91	56(20–82) ^#^	42	
Lasithiotakis 2016	United Kingdom	2004–2007	After evaluating the anastomosis	Single-center	Rectal cancer and benign disease	8(2) ^*^	Early	16	63(24) ^*^	10	1, 2, 3, 4, 5, 6, 8, 10
						57(38) ^*^	Intermediate	10	61(24) ^*^	5	
Danielson 2017 Park 2018 Keane 2019	Denmark and Sweden	2011–2015	After evaluating the anastomosis	Multicenter	Rectal cancer	11(8-152) ^#^	Early	55	67(36–82) ^#^	24	1, 2, 3, 4, 5, 6, 7, 8, 10, 11, 12, 13, 14
						148(64–665) ^#^	Late	57	67(39–81) ^#^	36	
Kłęk 2018	Poland	2016–2017	After evaluating the anastomosis	Multicenter	Rectal cancer	17.3 ± 1.5 ^^^	Early	29	55.7 ± 12.2 ^^^	18	1, 2, 3, 4, 5, 6, 7, 8, 9, 10, 11
						278.6 ± 89.1 ^^^	Late	29	56.2 ± 12.5 ^^^	16	
Gallyamov 2019	Russia	NR	After evaluating the anastomosis	Multicenter	Rectal cancer	11(8–21) ^#^	Early	31	62(32–78) ^#^	14	1, 2, 3, 4, 5, 7, 8, 10, 11
						148(64–265) ^#^	Late	34	67(35–77) ^#^	21	
Bausys 2019 Dulskas 2021	Lithuania	2011–2017	After evaluating the anastomosis	Single-center	Rectal cancer	34(29–47) ^*^	Early	43	65(56–72) ^*^	25	1, 2, 3, 4, 5, 6, 7, 8, 9, 10, 13, 14
						92(80–157) ^*^	Intermediate	38	66(60–70) ^*^	18	
Elsner 2021	Switzerland	2007–2014	Before evaluating the anastomosis	Multicenter	Rectal cancer	15(10–104) ^#^	Early	37	67(41–88) ^#^	21	1, 2, 3, 4, 5, 6, 8, 9, 10, 11, 14
						89(76–128) ^#^	Intermediate	34	67(48–87) ^#^	26	
Ellebæk 2023	Denmark	2011–2018	Before evaluating the anastomosis	Multicenter	Rectal cancer	47(30–63) ^$^	Intermediate	77	66.3 ± 8.1 ^^^	53	1, 2, 3, 4, 5, 10, 11, 14
						154(130–178) ^$^	Late	69	64.5 ± 9.1 ^^^	52	
Ahmadi-Amoli 2023	Iran	2016–2020	After evaluating the anastomosis	Single-center	Rectal cancer	64 ± 1 ^^^	Intermediate	50	63.2 ± 1.7 ^^^	29	1, 2, 3, 7, 10, 11, 12, 13, 14
						209 ± 2 ^^^	Late	54	63.2 ± 1.5 ^^^	33	
Vogel 2023	America	2018–2021	After evaluating the anastomosis	Multicenter	Ulcerative colitis	11(8–12) ^#^	Early	10	36(18–57) ^#^	5	1, 2, 3, 4, 5, 8, 9, 10
						59(45–182) ^#^	Intermediate	12	32(25–59) ^#^	10	

early closure: ≤ 35 days), intermediate closure: > 35 and ≤ 120 days, late closure: > 120 days

Outcomes: 1, total postoperative complications; 2, major postoperative complications; 3, mortality; 4, leak of rectal anastomosis; 5, bowel obstruction/ileus; 6, wound complications; 7, postoperative hospital stays; 8, reoperation; 9, readmission; 10, operative time; 11, blood loss; 12, stoma-related complications; 13, bowel function; 14, quality of life

*NR* not report, *IBD* inflammatory bowel disease, *FAP* familial adenomatous polyposis

^#^ median (range), ^*^ median (interquartile range), ^^^ mean ± SD, ^$^ mean (95% CI)

### Total postoperative complications

**Fig. 3:**
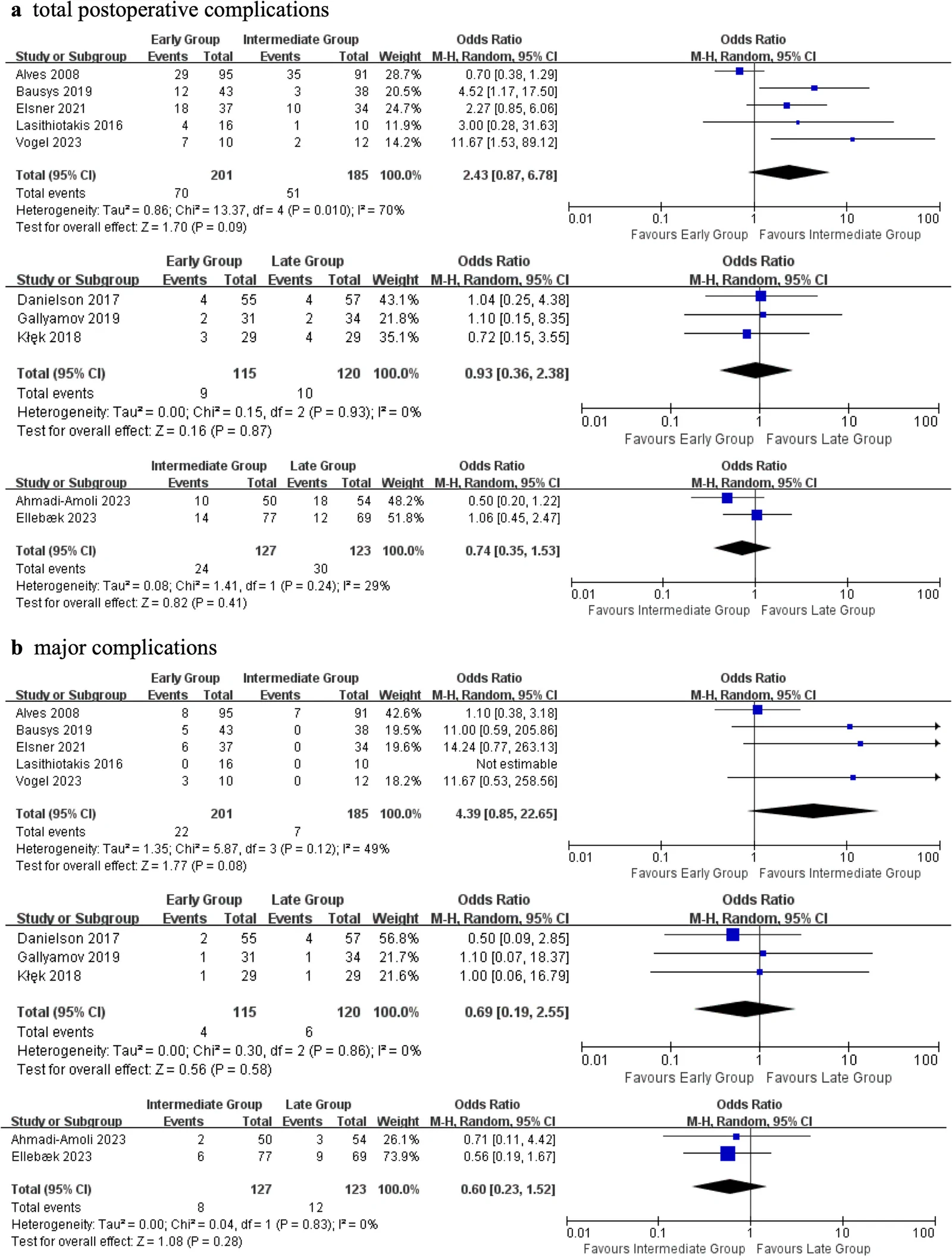
Direct comparison results of primary outcomes

Ten RCTs reported total postoperative complications [19–23, 43, 45, 46, 48, 49]. Overall, the rates of total postoperative complications in the early, intermediate, and late groups ranged from 6.5 to 70.0, 7.9–38.5, and 5.9–33.3%, respectively. Direct (Fig. 3a and Table S4) and network comparisons (Table 2 and Figure S2, GRADE low or moderate) showed no significant differences among the early, intermediate, and late groups.

**Table 2:** Network comparison results of the outcomes of interest

Outcomes	Articles (patients)	Comparison	Intermediate	Late
Total postoperative complications^#^	Early:8(316) Intermediate:7(312) Late:5(243)	Early	2.00 (0.93, 5.70)	1.20 (0.42, 3.60)
		Intermediate	-	0.59 (0.19, 1.60)
Major complications^#^	Early:8(316) Intermediate:7(312) Late:5(243)	Early	5.30 (1.30, 63.00)	1.50 (0.27, 12.00)
		Intermediate	-	0.29 (0.028, 1.50)
Leak of rectal anastomosis^#^	Early:8(316) Intermediate:6(262) Late:4(189)	Early	2.10 (0.38, 22.00)	2.70 (0.11, 72.00)
		Intermediate	-	1.20 (0.048, 22.00)
Bowel obstruction/ileus^#^	Early:8(316) Intermediate:7(312) Late:5(243)	Early	2.00 (0.92, 5.70)	1.20 (0.41, 3.70)
		Intermediate	-	0.59 (0.18, 1.60)
Wound complications^#^	Early:6(275) Intermediate:4(173) Late:2(86)	Early	4.80 (1.10, 35.00)	1.80 (0.20, 28.00)
		Intermediate	-	0.37 (0.021, 7.90)
Postoperative hospital stay^*^	Early:4(158) Intermediate:2(88) Late:4(174)	Early	0.50 (-0.51, 1.60)	0.55 (-0.45, 1.40)
		Intermediate	-	0.067 (-1.00, 0.91)
Reoperation^#^	Early:8(316) Intermediate:5(185) Late:3(120)	Early	11.00 (1.20, 4.1e + 02)	0.69 (0.034, 9.20)
		Intermediate	-	0.059 (0.00045, 1.70)
Readmission^#^	Early:4(119) Intermediate:3(84) Late:1(29)	Early	4.30 (0.43, 54.00)	2.6e + 06 (1.8e-19, 6.5e + 39)
		Intermediate	-	6.1e + 05 (3.7e-20, 1.6e + 39)
Operative time^*^	Early:8(316) Intermediate:7(312) Late:5(243)	Early	-4.20 (-14.00, 8.00)	-6.20 (-19.00, 6.40)
		Intermediate	-	-2.00 (-17.00, 10.00)
Blood loss^*^	Early:4(152) Intermediate:3(161) Late:5(243)	Early	-4.80 (-25.00, 16.00)	2.60 (-14.00, 20.00)
		Intermediate	-	7.50 (-9.70, 24.00)
Stoma-related complications^#^	Early:2(150) Intermediate:2(141) Late:2(111)	Early	0.086 (0.0057, 0.82)	0.076 (0.0066, 0.66)
		Intermediate	-	0.88 (0.093, 9.60)

^#^ odds ratio with 95% credible interval for binary outcome comparison. ^*^ weighted mean difference with 95% credible interval for continuous outcome comparison. “-” not applicable

### Major complications

Ten RCTs reported major complications [19–23, 43, 45, 46, 48, 49]. Overall, the rates of major complications in the early, intermediate, and late groups ranged from 0 to 16.2, 0-7.7, and 2.9–13.0%, respectively. Direct comparisons showed no significant differences in major complications among the early, intermediate, and late groups (Fig. 3b and Table S4). Regarding network meta-analysis (Table 2 and Figure S3), we found that compared with late closure, both early (OR 1.50, 95%CrI 0.27-12.00, GRADE moderate) and intermediate closure (OR 0.29, 95%CrI 0.028-1.50, GRADE moderate) had similar major complications; however, early closure had more major complications than intermediate closure (OR 5.30, 95%CrI 1.30–63.00, GRADE moderate). According to the SUCRA values, intermediate closure had the greatest advantage in reducing the rate of major complications (0.96), followed by late closure (0.38) and early closure (0.16).

### Secondary outcomes

Network comparison showed that compared with intermediate closure, early closure had more wound complications (OR 4.80, 95%CrI 1.10–35.00, GRADE high) and more reoperations (OR 11.0, 95%CrI 1.20–410, GRADE moderate), but fewer stoma-related complications (OR 0.086, 95%CrI 0.0057-0.82, GRADE moderate) (Table 2, Figure S6, S8, and S12). No significant differences among the early, intermediate, and late groups were identified for other outcomes (Table 2; Figures S7, S9, S10, and S11).

### Subgroup analyses

Results of subgroup analyses in studies on patients with rectal cancer, studies on patients after evaluating the integrity of the anastomosis, studies on patients with rectal cancer after evaluating the integrity of the anastomosis, studies on patients with low anterior resection or total mesorectal excision (LAR/TME) and studies on very early (≤ 15 days), intermediate, and late closure are summarized in Table 3 and S7, and are described fully in the Supplementary Results.

**Table 3:** Network comparison results of subgroup analysis

Subgroup analysis	Articles (patients)	Comparison	Intermediate	Late
Rectal cancer				
Total postoperative complications	Early:5(195) Intermediate:4(199) Late:5(243)	Early	2.30 (0.95, 5.70)	1.30 (0.53, 3.30)
		Intermediate	-	0.59 (0.24, 1.30)
Major complications	Early:5(195) Intermediate:4(199) Late:5(243)	Early	7.90 (1.20, 1.8e + 02)	1.90 (0.31, 18.00)
		Intermediate	-	0.24 (0.019, 1.30)
Time points of randomization (after evaluating the integrity of the anastomosis)				
Total postoperative complications	Early:7(279) Intermediate:5(201) Late:4(174)	Early	2.40 (0.83, 9.50)	0.99 (0.26, 4.20)
		Intermediate	-	0.42 (0.077, 1.80)
Major complications	Early:7(279) Intermediate:5(201) Late:4(174)	Early	3.90(0.79, 54.00)	1.00 (0.16, 8.90)
		Intermediate	-	0.26 (0.017, 2.10)
After evaluating the integrity of the anastomosis in rectal cancer				
Total postoperative complications	Early:4(158) Intermediate:2(88) Late:4(174)	Early	3.10 (0.85, 13.00)	1.20 (0.38, 3.40)
		Intermediate	-	0.37 (0.098, 1.30)
Major complications	Early:4(158) Intermediate:2(88) Late:4(174)	Early	6.40 (0.48, 2.6e + 02)	1.20 (0.17, 10.00)
		Intermediate	-	0.18 (0.0067, 2.40)
Very early vs. intermediate vs. late closure				
Total postoperative complications	Early:6(244) Intermediate:6(274) Late:4(214)	Very early	1.80 (0.73, 6.30)	1.20 (0.35, 5.10)
		Intermediate	-	0.67 (0.18, 2.20)
Major complications	Early:6(244) Intermediate:6(274) Late:4(214)	Very early	3.70 (0.78, 49.00)	1.30 (0.20, 15.00)
		Intermediate	-	0.36 (0.034, 2.20)
Very early vs. intermediate vs. late closure in rectal cancer				
Total postoperative complications	Early:3(123) Intermediate:3(161) Late:4(214)	Very early	2.00 (0.72, 5.30)	1.30 (0.49, 3.60)
		Intermediate	-	0.68 (0.31, 1.50)
Major complications	Early:3(123) Intermediate:3(161) Late:4(214)	Very early	4.90 (0.44, 1.7e + 02)	1.60 (0.17, 23.00)
		Intermediate	-	0.32 (0.026, 2.30)
Very early vs. intermediate vs. late closure after evaluating the integrity of the anastomosis				
Total postoperative complications	Early:5(207) Intermediate:4(163) Late:3(145)	Very early	2.10 (0.56, 12.00)	1.00 (0.19, 6.90)
		Intermediate	-	0.50 (0.062, 3.00)
Major complications	Early:5(207) Intermediate:4(163) Late:3(145)	Very early	2.10 (0.38, 28.00)	0.84 (0.11, 8.60)
		Intermediate	-	0.39 (0.026, 3.60)
Very early vs. intermediate vs. late closure after evaluating the integrity of the anastomosis in rectal cancer				
Total postoperative complications	Early:2(86) Intermediate:1(50) Late:3(145)	Very early	2.20 (0.37, 13.00)	1.10 (0.28, 4.10)
		Intermediate	-	0.50 (0.15, 1.60)
Major complications	Early:2(86) Intermediate:1(50) Late:3(145)	Very early	0.90 (0.063, 13.00)	0.62 (0.11, 3.10)
		Intermediate	-	0.69 (0.077, 5.10)
Rectal cancer subgroup (LAR/TME cohort)				
Total postoperative complications	Early:3(121) Intermediate:3(161) Late:4(209)	Early	0.90 (0.063, 13.00)	0.62 (0.11, 3.10)
		Intermediate	-	0.69 (0.077, 5.10)
Major complications	Early:3(121) Intermediate:3(161) Late:4(209)	Early	5.59 (0.43, 148.30)	1.66 (0.17, 22.04)
		Intermediate	-	0.30 (0.025, 2.25)

If the patient was randomized after evaluating the integrity of the anastomosis (without clinical or radiological signs of anastomotic leakage), the total postoperative and major complications and leak of rectal anastomosis were all similar between early and intermediate closure, between early and late closure, between very early and intermediate closure, and between very early and late closure. Besides, for subgroup analysis in studies on patients after evaluating the integrity of the anastomosis, early closure had fewer stoma-related complications than intermediate (OR 0.086, 95%CrI 0.0060–0.81, GRADE moderate) and late closure (OR 0.076, 95%CrI 0.0073-0.65, GRADE moderate); very early closure had more wound complications than late closure (OR 3.2e + 07, 95%CrI 3.60-2.0e + 25, GRADE moderate), and very early closure had fewer stoma-related complications than intermediate (OR 0.087, 95%CrI 0.0059-0.87, GRADE moderate) and late closure (OR 0.076, 95%CrI 0.0072-0.63, GRADE moderate). For subgroup analysis in studies on patients with rectal cancer after evaluating the integrity of the anastomosis, early closure had more wound complications (OR 4.8e + 08, 95%CrI 5.00-3.3e + 28, GRADE moderate) than intermediate closure. For subgroup analysis in studies on patients with LAR/TME, network comparison showed no evidence of a significant difference between early, intermediate, and late closures for total postoperative complications and major complications.

### Publication bias

Publication bias was assessed based on the total postoperative and major complications (Figure S29). Visual inspection of the adjusted funnel plot revealed minor asymmetry among the studies, indicating a potential publication bias.

## Discussion

The optimal timing of defunctioning ileostomy closure remains unclear. We conducted this Bayesian network meta-analysis to compare early, intermediate, and late ileostomy closures. Direct and indirect evidence from 10 RCTs were combined, and we found that compared with intermediate and late closure, early closure of ileostomy can be considered in selected patients without clinical or radiological signs of anastomotic leakage, with similar total postoperative and major complications, and early closure had fewer stoma-related complications but more wound complications.

From a physiological perspective, temporary diversion functions as a protective adjunct during the early phase of anastomotic healing. Thus, as long as the rectal anastomosis heals, the ileostomy can, theoretically, be closed. Although there are no data available concerning the healing process of colorectal anastomoses in humans, experimental data derived from non-human primates suggest a rapid increase in anastomotic tensile strength after postoperative day five, with mechanical integrity exceeding baseline levels by day seven [50]. Moreover, most anastomotic leaks are reported to be present within ten days of rectal surgery [51]. Therefore, early closure of the ileostomy is potentially feasible, as long as anastomotic healing is confirmed.

Several trials have assessed early ileostomy closure. The first RCT conducted by Alves et al.[20] reported feasibility of early closure without excess morbidity in selected patients, findings corroborated by several subsequent RCTs [19, 45]. However, three RCTs were terminated prematurely because early closure increased postoperative complications [21–23]. A closer inspection of these studies suggests that heterogeneity in trial design and patient selection likely underpins these discrepancies. For example, Elsner et al. [21]randomized participants before evaluating the anastomosis integrity, introducing potential contamination of the early-closure cohort with subclinical leaks. In Bausys et al.[22], the median interval between primary surgery and closure was 34 days, coinciding with a period in which adhesion maturation may progress and increase operative difficulty [52]; however, this mechanism remains inferential rather than directly demonstrated. Similarly, disease-specific inclusion criteria, such as ulcerative colitis in Vogel et al.[23], may have independently influenced postoperative risk profiles, complicating cross-trial comparability [53].

Against this heterogeneous backdrop, our network meta-analysis demonstrated no statistically significant differences in total postoperative complications across early, intermediate, and late closure strategies. Although major complications were numerically higher after early closure compared with intermediate closure in the primary analysis, this signal attenuated in the subgroup restricted to patients randomized after confirmation of anastomotic integrity. Importantly, no increase in rectal anastomotic leak was observed with early closure compared with intermediate or late strategies, consistent with several prior meta-analyses [24, 25, 27]. By contrast, one recent synthesis reported higher leak rates following early closure [28]; however, its outcome definition incorporated heterogeneous entities, including intra-abdominal abscess and enterocutaneous fistula, potentially inflating event rates and limiting comparability.

A more consistent finding emerged for wound-related morbidity. Early closure was associated with an increased risk of wound complications compared with intermediate closure, a result aligned with prior pooled analyses [24, 25]. Although technical refinements such as purse-string skin closure have demonstrated reductions in surgical site infection after ileostomy closure [54, 55], whether these benefits extend to early closure populations remains uncertain, as procedural variables were incompletely reported across included trials and could not be formally assessed.

Taken together, the evidence does not support a binary interpretation of early closure as uniformly safe or hazardous. Rather, its effects appear domain-specific: early reversal may reduce stoma-related morbidity associated with prolonged diversion, while simultaneously increasing susceptibility to wound-related complications. Clinical decision-making should therefore be individualized, integrating anastomotic integrity, patient physiological reserve, stoma-related burden, and readiness for reoperation rather than adhering to a fixed temporal threshold.

When situated within prior pairwise meta-analyses, our findings extend the literature by challenging the conventional dichotomy between early and delayed closure. Most earlier syntheses collapsed intermediate and late closure into a single comparator. Farag et al.[56] found that reversal within 2 weeks after distal colorectal resection was feasible, without an apparent increase in anastomotic leakage, overall postoperative morbidity, operative time, or length of stay compared with delayed closure. Cheng et al.[25] incorporating trial sequential analysis, reported lower risks of small-bowel obstruction/postoperative ileus and shorter total operative time after early closure, but also a higher incidence of surgical site infection; the evidence for overall morbidity, reoperation, and anastomotic leakage remained weak. Chan et al.[57] focusing specifically on rectal cancer, similarly found a higher risk of surgical complications after early closure, without significant differences in overall morbidity, reoperation, or anastomotic leakage. By explicitly modelling three discrete timing strata, our analysis suggests that intermediate closure may represent a clinically meaningful transitional phase rather than a residual category within delayed reversal. Early closure did not differ materially from late closure for most outcomes; however, intermediate closure ranked most favorably for major complications in probabilistic analyses, a finding that should be interpreted cautiously given the uncertainty inherent in several network estimates.

Whether these patterns generalize to rectal cancer populations remains uncertain. To address this question, we separately analyzed patients undergoing low anterior resection with total mesorectal excision, a cohort characterized by oncologic pelvic dissection, frequent neoadjuvant therapy, and low colorectal anastomosis. This stratification reduced clinical heterogeneity and improved interpretability of timing effects. Moreover, as total neoadjuvant therapy becomes more widely adopted, the conventional need to defer stoma reversal until completion of postoperative systemic therapy may become less compelling in selected patients [24, 57]. Within this subgroup, no statistically credible differences were observed among timing strategies, suggesting attenuation of signals detected in the broader pooled population; however, this should not be interpreted as equivalence, but rather as limited statistical resolution.

Beyond surgical outcomes, prolonged ileostomy exposure imposes measurable burdens on QoL and bowel function. Consistent with this, one multicenter RCT reported a substantially higher incidence of stoma-related complications after late closure than after early closure (77% vs. 24%)[19], and our network meta-analysis similarly found that early closure reduced stoma-related complications, which was in agreement with a previous meta-analysis [24]. However, improvements in validated QoL instruments, including the GIQLI [20, 21, 48], SF-36[47, 49], EORTC QLQ-C30[21, 44, 47] and EORTC QLQ-CR29[47] were not consistently demonstrated across trials; only one study reported worse scores in the late-closure group on the urgency/soiling subscale of the Memorial Sloan Kettering Cancer Center Bowel Function Instrument (MSKCC BFI) [17]. Evidence for bowel function outcomes, including LARS, also remained heterogeneous. The LARS scores was comparable among the early, intermediate, and late closure groups in three trials [17, 44, 49], and in one meta-analysis [24], whereas two other meta-analyses suggested that a prolonged time to closure may adversely affect major LARS [18, 58]. Divergent findings likely reflect variability in closure timing definitions, follow-up intervals, and outcome reporting rather than true biological inconsistency. As a result, robust conclusions regarding functional recovery cannot be drawn from the current evidence base.

To the best of our knowledge, this is the first network meta-analysis comparing different time intervals for ileostomy closure after rectal surgery. Our study integrated all the current evidence from RCTs and highlighted the feasibility and safety of early closure in selected patients. Nevertheless, several limitations should be acknowledged. First, 60% of included RCTs enrolled fewer than 50 patients per group, increasing susceptibility to small-study effects. Second, clinical heterogeneities persisted with respect to primary bowel disease, timing of randomization, and the wide distribution of early-closure intervals, which ranged from 8 to 34 days. Although subgroup analyses were performed to assess robustness, several comparisons remained underpowered. Third, the surgical approach used for the index rectal resection (open, laparoscopic, robotic, or other minimally invasive techniques) represents a potential source of clinical heterogeneity, because approach-specific differences may influence adhesions, wound morbidity, postoperative recovery, and patient selection. However, most included RCTs either enrolled mixed surgical populations or did not report closure outcomes separately by index surgical approach. A formal subgroup analysis was therefore unreliable, as only two trials clearly reported predominantly minimally invasive approaches and separate outcome data were unavailable. Fourth, defunctioning ileostomy is commonly used in rectal cancer populations receiving neoadjuvant or adjuvant therapy, but treatment-specific subgroup analyses could not be performed because of limited reporting. Finally, bowel function and QoL were incompletely and inconsistently reported, precluding quantitative synthesis and raising the possibility of reporting bias. Regardless of these limitations, our network meta-analysis provides evidence to support the use of early closure in selected patients.

In conclusion, the current network meta-analysis suggests that early closure may be feasible in carefully selected patients without clinical or radiological evidence of anastomotic leakage, yielding comparable overall and major postoperative morbidity while reducing stoma-related complications but increasing wound-related events. Further prospective studies are required to validate the efficacy of early closure.

## Supplementary Information

Below is the link to the electronic supplementary material.

Supplementary Material 1 (DOCX 10.0 MB)

## Data Availability

The data used in this study were derived from published randomized controlled trials and are available within the article and its supplementary materials. No new individual participant data were generated during this study. Any additional data supporting the findings of this study are available from the corresponding authors upon reasonable request.
