# Supplementary material for: Optimal timing of ileostomy closure after rectal surgery: A Bayesian network meta-analysis of randomized controlled trials
Source: Langenbecks Arch Surg. 2026 Jul 9;411(1):259. doi: 10.1007/s00423-026-04133-z (PMC13631017; doi:10.1007/s00423-026-04133-z)
Supplement: Supplementary file 1 [file 423_2026_4133_MOESM1_ESM.docx]

**Optimal timing of ileostomy closure after rectal surgery: a Bayesian network meta-analysis of randomized controlled trials**

**Authors:**

Zikai Zhou^1,2^, Siyi Wang^1,2^, Jiahao Zhou^1,2^, Jun Huang^1,2^, Xiangbing Deng^1^, Ziqiang Wang^1^, Wenjian Meng^1,#^, Qingbin Wu^1,#^

^1^ Colorectal Cancer Center, Department of General Surgery, West China Hospital, Sichuan University, Chengdu 610041, China

^2^ West China School of Medicine, Sichuan University, Chengdu 610041, China.

^#^ Co-corresponding authors:

Dr. Qingbin Wu, Colorectal Cancer Center, Department of General Surgery, West China Hospital, Sichuan University, No. 37, Guo Xue Xiang, Chengdu, China. Phone: +8602885422480. E-mail address: wuqingbin214@scu.edu.cn. ORCID: 0000-0001-8149-5989

Dr. Wenjian Meng, Colorectal Cancer Center, Department of General Surgery, West China Hospital, Sichuan University, No. 37, Guo Xue Xiang, Chengdu, China. Phone: +8602885422480. E-mail address: mengwenjian@126.com

**Supplementary Methods**

***Study selection***

The search items were “ileostomy OR ileostomies” AND “randomized OR clinical trial OR clinical trials OR RCT OR RCTs” (Table S1).

***Inclusion and exclusion criteria***

Studies were included if they met the following criteria: (1) Participants: Patients who underwent rectal surgery with a primary low colorectal, coloanal, or ileoanal anastomosis and creation of a temporary diverting ileostomy. Eligible populations included patients undergoing surgery for rectal cancer or other benign or malignant rectal diseases, provided that the index operation involved rectal resection with temporary ileostomy formation. (2) Interventions: Early ileostomy closure after the index rectal surgery. In the present network meta-analysis, closure timing was categorized according to the median or mean interval between the primary operation and ileostomy closure, with early closure defined as ≤35 days. (3) Comparator: Standard or delayed ileostomy closure. According to the predefined network structure, comparator groups were classified as intermediate closure (>35 and ≤120 days) and late closure (>120 days) based on the median or mean interval from primary rectal surgery to ileostomy closure. (4) Outcomes: Studies were required to report at least one clinically relevant postoperative outcome after ileostomy closure. The primary outcomes were total postoperative complications and major complications. Secondary outcomes included anastomotic leak, bowel obstruction or ileus, wound complications, stoma-related complications, reoperation, readmission, operative time, blood loss, postoperative length of hospital stay, bowel function, and quality of life. (5) Study Design: Randomized controlled trials comparing different timing strategies for temporary ileostomy closure were eligible for inclusion. When multiple publications reported data from the same randomized trial, they were collated and treated as a single trial, with the most complete and relevant dataset used for each outcome. The exclusion criteria were as follows: (1) studies that were not RCT, (2) studies that included patients with defunctioning colostomies, (3) studies that did not report any of the outcomes below, and (4) studies that were reviews, meta-analyses, conference abstracts, comments, or letters.

***Data extraction and outcomes***

Two investigators (Zhou Z and Zhou J) independently screened all identified items and extracted the characteristics and outcomes of the included studies. Disagreements were resolved with the assistance of a third investigator (Wu Q). The following data were extracted from each RCT: first author’s name, year of publication, country, registration ID, study period, time point of randomization, patient eligibility, primary bowel disease, index operation, time interval between primary surgery and ileostomy closure, sample size, male sex, age, body mass index, tumor location, neoadjuvant therapy, and outcomes.

The primary outcomes were total and major postoperative complications. Major complications were defined as grade 3a or higher according to the Clavien-Dindo classification system^1^. Secondary outcomes included operative time, blood loss, mortality, anastomotic leak, bowel obstruction, ileus, wound complications, postoperative hospital stay, reoperation, readmission, bowel function, quality of life, and stoma-related complications. Stoma-related complications were defined as any adverse events directly attributable to the presence or reversal of a temporary ileostomy. Based on the definitions used in the included randomized controlled trials, these included: (1) local stoma site complications (e.g., peristomal dermatitis, infection, bleeding, mucocutaneous separation), (2) structural complications (e.g., prolapse, retraction, stenosis, parastomal hernia), (3) functional complications (e.g., high-output stoma, dehydration, obstruction), and (4) stoma-related clinical events, including readmission or reoperation due to stoma dysfunction or complications after stoma closure.

***Quality assessment***

The standard Jadad scoring system and the Cochrane Risk Assessment Tool were used to assess the quality of the enrolled RCTs simultaneously by two investigators (Zhou Z and Zhou J) independently, and disagreements were resolved with the assistance of a third investigator (Wu Q). The standard Jadad scoring system includes three aspects with a total score of five: randomization, blinding, and inclusion of all patients (1–2 scores, low quality; 3–5 scores, high quality) ^2^. The Cochrane Risk Assessment Tool focused on bias in selection, performance, detection, attrition, reporting, and other, and each bias was evaluated as low, unclear, or high risk ^3^.

**Data synthesis and analysis**

All direct and indirect comparisons of each outcome were synthesized and reported as odds ratios (OR) for binary outcomes and weighted mean difference (WMD) for continuous outcomes, along with the corresponding 95% confidence intervals (95%CI) or credible intervals (95%CrIs). For continuous data, mean and standard deviation (SD) were extracted directly from the included trials or calculated according to the formula proposed by Hozo and colleagues ^4^.

A direct meta-analysis with a random-effects model was conducted to calculate the OR/WMD with 95%CI for each pairwise comparison between the early, intermediate, and late groups. The software Review Manager (version 5.3, Cochrane Collaboration, Copenhagen, Denmark) was used for all syntheses, and a P value <0.05 was considered statistically significant.

A random-effects Bayesian network meta-analysis using the Markov chain Monte Carlo methods and uninformative priors was conducted for each outcome ^5, 6^. The detailed analysis methods were described in our recent published work ^7^. Briefly, a network plot was drawn to show direct and indirect comparisons among the three groups. Four chains were selected to fit the model with 100000 simulation iterations after a burn-in of 50000 iterations and a thinning interval of 10 for each chain to achieve convergence of the model. The convergence and trajectory density diagrams were constructed to evaluate the convergence and stability of the models, respectively. The node-splitting method was used to evaluate the network consistency by comparing the direct and indirect estimates ^8^. If the difference in DIC values between the consistency and inconsistency models was not significant, the consistency model was applied; otherwise, the inconsistency model was selected. Direct and indirect comparisons was combined to estimate the outcome, reported as OR/WMD with 95%CrI. The surface under the cumulative ranking curve (SUCRA) was calculated to show the rank probability of these the groups, which ranged from 0 (worst) to 1 (best) ^9^. A funnel plot was drawn to evaluate publication bias based on primary outcomes. The Grading of Recommendations Assessment, Development and Evaluation (GRADE) criteria for network meta-analysis was used to evaluate the quality of evidence ^10^. The R software (version 3.6.2, Foundation for Statistical Computing, Vienna, Austria) with gemtc package (version 1.0-1) was used for all Bayesian network meta-analysis ^11, 12^, and a P value <0.05 was considered statistically significant.

To evaluate the robustness and reliability of the results, we conducted several subgroup analyses. The first analysis stratified the included studies according to the primary bowel disease (rectal cancer or other diseases) to observe the influence of different diseases. The second analysis stratified the included studies according to the time point of randomization (before and after evaluating the integrity of the anastomosis). The third analysis compared very early (≤15 days), intermediate, and late closure to assess the effect of closure at an earlier time. The fourth analysis compared patients with low anterior resection or total mesorectal excision (LAR/TME).

**Supplementary Results**

***Study selection***

A flowchart of this study is shown in Figure 1. After duplicates were discarded, 3851 records were identified. After screening the titles and abstracts, 56 studies were evaluated. Of these, 43 were excluded, three had insufficient data, one included patient who underwent colostomy, ten were conference abstracts, three were letters or comments, 18 were reviews or meta-analysis, and nine were case–control studies but not RCT. Finally, thirteen publications representing 10 RCTs were included in our network meta-analysis ^13-25^.

***Study characteristics***

The characteristics of the included studies are summarized in Tables 1 and S2. Ten studies from seven RCTs included only patients with rectal cancer ^15, 16, 18-25^, one RCT included only patients with ulcerative colitis ^17^, and two RCTs included patients with mixed rectal cancer and benign disease ^13, 14^. According to the time point of randomization, eleven studies from eight RCTs randomized participants after evaluating the integrity of the anastomosis ^13-15, 17-23, 25^, while the other two RCTs randomized participants before evaluating the anastomosis ^16, 24^. For early closure, eight studies from six RCTs included patients with a median or mean time interval between primary surgery and closure ≤ 15 days (very early group) ^13, 14, 16, 17, 19, 21-23^, while three studies from two RCTs included patients with time interval > 15 and ≤ 35 days ^15, 18, 20^.

***Quality assessment of included RCTs***

The risk of bias of the included RCTs is summarized in Table S3 and Figure S1. All 10 RCTs were high quality trials (3 scores) according to the standard Jadad scoring system. Besides, the Cochrane Risk Assessment Tool indicated that the overall quality was adequate, except with regard to the blinding of participants and personnel (performance bias) and other bias.

***Leak of rectal anastomosis***

Nine RCTs reported leak of rectal anastomosis ^13-17, 19-21, 24^. Overall, the rates of leak of rectal anastomosis after closure in the early, intermediate, and late groups ranged from 0–8.1, 0–8.8, and 0–1.8%, respectively. Direct (Table S4) and network comparisons (Table 2 and Figure S4, GRADE *low or moderate*) showed no significant differences among the early, intermediate, and late groups.

***Bowel obstruction/ileus***

Nine RCTs reported bowel obstruction/ileus ^13-17, 19-21, 24^. Overall, the rates of bowel obstruction/ileus in the early, intermediate, and late group ranged from 1.8–50.0, 5.3–16.7, and 0–2.9%, respectively. Direct (Table S4) and network comparisons (Table 2 and Figure S5, GRADE *low or moderate*) showed no significant differences among the early, intermediate, and late groups.

***Wound complications***

Six RCTs reported wound complications ^13-16, 19, 20^. Overall, the wound complication rates in the early, intermediate, and late groups ranged from 3.6–18.9, 0–5.9, and 0–10.3%, respectively. Direct comparisons showed that early closure had more wound complications than intermediate closure (OR 3.36, 95%CI 1.46-7.74), while early and late closure had similar wound complications (Table S4). Network comparisons showed that compared with late closure, both early (OR 1.80, 95%CrI 0.20–28.00, GRADE *moderate*) and intermediate closure (OR 0.37, 95%CrI 0.021–7.90, GRADE *low*) had similar wound complications; however, early closure had more wound complications than intermediate closure (OR 4.80, 95%CrI 1.10–35.00, GRADE *high*). According to the SUCRA values, intermediate closure had the best advantage in reducing the rate of wound complications (0.88), followed by late closure (0.47) and early closure (0.16).

***Other secondary outcomes***

Network comparison showed that early closure had more reoperations (OR 11.0, 95%CrI 1.20–4.1e+02, GRADE *moderate*) but fewer stoma-related complications (OR 0.086, 95%CrI 0.0057–0.82, GRADE *moderate*) than intermediate closure (Table 2, Figure S8, and S12); besides, early closure also had fewer stoma-related complications (OR 0.076, 95%CrI 0.0066–0.66, GRADE *moderate*) than late closure (Table 2 and Figure S12). No significant differences between the early, intermediate, and late groups were identified for the other endpoints (Table 2, Figures S7, S9, S10, and S11).

Three RCTs reported bowel function assessed using the LARS questionnaire (Table S5) ^18, 23, 25^, while three reported quality of life assessed using the Gastrointestinal Quality of Life Index (GIQLI) questionnaire (Table S6) ^13, 16, 24^. The LARS and GIQLI scores were comparable among the early, intermediate, and late closure groups in each trial. However, we could not perform direct or network comparisons because LARS and GIQLI were assessed at different follow-up times.

***Subgroup analyses***

Several subgroup analyses were performed to evaluate the robustness and reliability of the results (Tables 3 and S7).

*Rectal cancer*

Seven RCTs included only patients with rectal cancer ^15, 16, 19-21, 24, 25^. Network comparisons showed no evidence of a significant difference between early, intermediate, and late closures in terms of total postoperative complications (Table 3 and Figure S13, GRADE *moderate*). Compared with late closure, both early and intermediate closures had similar major complications (GRADE *low*); however, early closure had more major complications than intermediate closure (OR 7.90, 95%CrI 1.20–1.8e+02, GRADE *high*) (Table 3 and Figure S14). According to the SUCRA values, intermediate closure had the best advantage in reducing the rate of major complications (0.97), followed by late closure (0.41) and early closure (0.12).

*Time points of randomization (after evaluating the integrity of the anastomosis)*

There were 8 RCTs that randomized participants after evaluating the integrity of the anastomosis ^13-15, 17, 19-21, 25^. Network comparisons showed no evidence of a significant difference between early, intermediate, and late closures in terms of total postoperative complications (Table 3 and Figure S15, GRADE *low or moderate*) and major complications (Table 3 and Figure S16, GRADE *low or moderate*).

*After evaluating the integrity of the anastomosis in rectal cancer*

There were 5 RCTs that only included patients with rectal cancer and randomized participants after evaluating the integrity of the anastomosis ^15, 19-21, 25^. Network comparison showed no evidence of a significant difference between early, intermediate, and late closures in terms of total postoperative complications (Table 3 and Figure S17, GRADE *moderate*) and major complications (Table 3 and Figure S18, GRADE *low*).

*Very early vs intermediate vs late closure*

There were 8 RCTs that compared very early, intermediate, and late closure ^13, 14, 16, 17, 19, 21, 24, 25^. Network comparisons showed no evidence of a significant difference between early, intermediate, and late closures in terms of total postoperative complications (Table 3 and Figure S19, GRADE *low or* *moderate*) and major complications (Table 3 and Figure S20, GRADE *low or* *moderate*).

*Very early vs intermediate vs late closure in rectal cancer*

There were 5 RCTs that compared very early, intermediate, and late closures in patients with rectal cancer ^16, 19, 21, 24, 25^. Network comparison showed no evidence of a significant difference between early, intermediate, and late closures in terms of total postoperative complications (Table 3 and Figure S21, GRADE *moderate*) and major complications (Table 3 and Figure S22, GRADE *low*).

*Very early vs intermediate vs late closure after evaluating the integrity of the anastomosis*

There were 6 RCTs that compared very early, intermediate, and late closures after evaluating the integrity of the anastomosis ^13, 14, 17, 19, 21, 25^. Network comparisons showed no evidence of a significant difference between early, intermediate, and late closures in terms of total postoperative complications (Table 3 and Figure S23, GRADE *low or moderate*) and major complications (Table 3 and Figure S24, GRADE *moderate*).

*Very early vs intermediate vs late closure after evaluating the integrity of the anastomosis in rectal cancer*

There were 3 RCTs that compared very early, intermediate, and late closures after evaluating the integrity of the anastomosis in rectal cancer ^19, 21, 25^. Network comparison showed no evidence of a significant difference between early, intermediate, and late closures for total postoperative complications (Table 3 and Figure S25, GRADE *low*) and major complications (Table 3 and Figure S26, GRADE *low*).

*Rectal cancer subgroup (LAR/TME cohort)*

There were 5 RCTs that compared early, intermediate, and late closure strategies in the rectal cancer subgroup (LAR/TME cohort)^16, 19, 20, 24, 25^. Network comparison showed no evidence of a significant difference between early, intermediate, and late closures for total postoperative complications (Table 3 and Figure S27, GRADE *low*) and major complications (Table 3 and Figure S28, GRADE *low*).

**Reference**

1	Dindo D, Demartines N, Clavien PA. Classification of surgical complications: a new proposal with evaluation in a cohort of 6336 patients and results of a survey. Ann Surg 2004; 240 (2): 205-213.

2	Jadad AR, Moore RA, Carroll D et al. Assessing the quality of reports of randomized clinical trials: is blinding necessary? Control Clin Trials 1996; 17 (1): 1-12.

3	Higgins JP, Altman DG, Gøtzsche PC et al. The Cochrane Collaboration's tool for assessing risk of bias in randomised trials. Bmj 2011; 343: d5928.

4	Hozo SP, Djulbegovic B, Hozo I. Estimating the mean and variance from the median, range, and the size of a sample. BMC Med Res Methodol 2005; 5: 13.

5	Dias S, Sutton AJ, Ades AE et al. Evidence synthesis for decision making 2: a generalized linear modeling framework for pairwise and network meta-analysis of randomized controlled trials. Med Decis Making 2013; 33 (5): 607-617.

6	Lu G, Ades AE. Combination of direct and indirect evidence in mixed treatment comparisons. Stat Med 2004; 23 (20): 3105-3124.

7	Wu Q, Zhou J, Huang J et al. Total neoadjuvant therapy versus chemoradiotherapy for locally advanced rectal cancer: Bayesian network meta-analysis. Br J Surg 2023; 110 (7): 784-796.

8	van Valkenhoef G, Dias S, Ades AE et al. Automated generation of node-splitting models for assessment of inconsistency in network meta-analysis. Res Synth Methods 2016; 7 (1): 80-93.

9	Salanti G, Ades AE, Ioannidis JP. Graphical methods and numerical summaries for presenting results from multiple-treatment meta-analysis: an overview and tutorial. J Clin Epidemiol 2011; 64 (2): 163-171.

10	Puhan MA, Schünemann HJ, Murad MH et al. A GRADE Working Group approach for rating the quality of treatment effect estimates from network meta-analysis. Bmj 2014; 349: g5630.

11	van Valkenhoef G, Lu G, de Brock B et al. Automating network meta-analysis. Res Synth Methods 2012; 3 (4): 285-299.

12	Viechtbauer W. Conducting Meta-Analyses in R with the metafor Package. J Stat Softw 2010; 36 (3): 1 - 48.

13	Alves A, Panis Y, Lelong B et al. Randomized clinical trial of early versus delayed temporary stoma closure after proctectomy. British Journal of Surgery 2008; 95 (6): 693-698.

14	Lasithiotakis K, Aghahoseini A, Alexander D. Is Early Reversal of Defunctioning Ileostomy a Shorter, Easier and Less Expensive Operation? World J Surg 2016; 40 (7): 1737-1740.

15	Bausys A, Kuliavas J, Dulskas A et al. Early versus standard closure of temporary ileostomy in patients with rectal cancer: A randomized controlled trial. J Surg Oncol 2019; 120 (2): 294-299.

16	Elsner AT, Brosi P, Walensi M et al. Closure of Temporary Ileostomy 2 Versus 12 Weeks After Rectal Resection for Cancer: A Word of Caution From a Prospective, Randomized Controlled Multicenter Trial. Dis Colon Rectum 2021; 64 (11): 1398-1406.

17	Vogel JD, Fleshner PR, Holubar SD et al. High Complication Rate After Early Ileostomy Closure: Early Termination of the Short Versus Long Interval to Loop Ileostomy Reversal After Pouch Surgery Randomized Trial. Dis Colon Rectum 2023; 66 (2): 253-261.

18	Dulskas A, Petrauskas V, Kuliavas J et al. Quality of Life and Bowel Function Following Early Closure of a Temporary Ileostomy in Patients with Rectal Cancer: A Report from a Single-Center Randomized Controlled Trial. J Clin Med 2021; 10 (4).

19	Danielsen AK, Park J, Jansen JE et al. Early Closure of a Temporary Ileostomy in Patients With Rectal Cancer: A Multicenter Randomized Controlled Trial. Ann Surg 2017; 265 (2): 284-290.

20	Kłęk S, Pisarska M, Milian-Ciesielska K et al. Early closure of the protective ileostomy after rectal resection should become part of the Enhanced Recovery After Surgery (ERAS) protocol: a randomized, prospective, two-center clinical trial. Wideochir Inne Tech Maloinwazyjne 2018; 13 (4): 435-441.

21	Gallyamov EA, Agapov MA, Lutsevich OE et al. Early ileostomy closure in patients with rectal cancer. Primary results of the randomized controlled multicenter trial. Khirurgiia (Mosk) 2019 (6): 35-40.

22	Park J, Danielsen AK, Angenete E et al. Quality of life in a randomized trial of early closure of temporary ileostomy after rectal resection for cancer (EASY trial). Br J Surg 2018; 105 (3): 244-251.

23	Keane C, Park J, Öberg S et al. Functional outcomes from a randomized trial of early closure of temporary ileostomy after rectal excision for cancer. Br J Surg 2019; 106 (5): 645-652.

24	Ellebæk MB, Perdawood SK, Steenstrup S et al. Early versus late reversal of diverting loop ileostomy in rectal cancer surgery: a multicentre randomized controlled trial. Sci Rep 2023; 13 (1): 5818.

25	Ahmadi-Amoli H, Rahimi M, Abedi-Kichi R et al. Early closure compared to late closure of temporary ileostomy in rectal cancer: a randomized controlled trial study. Langenbecks Arch Surg 2023; 408 (1): 234.

Table S1. Search strategy of the systematic review.

| Database and search strategy | | Items |
| --- | --- | --- |
| **PubMed (up to June 21, 2026)** | | |
| 1 | ileostomy[MeSH Terms] OR ileostomy OR ileostomies | 12442 |
| 2 | randomized controlled trial[MeSH Terms] OR randomized OR clinical trial OR clinical trials OR RCT OR RCTs | 2652646 |
| 3 | 1 AND 2 | 911 |
| **Web of Science Core Collection (1900-June 21, 2026)** | | |
| 1 | ALL=(ileostomy OR ileostomies) | 9064 |
| 2 | ALL=(randomized OR clinical trial OR clinical trials OR RCT OR RCTs) | 2128562 |
| 3 | 1 AND 2 | 981 |
| **Embase (via Ovid, 1974 to June 21, 2026)** | | |
| 1 | (ileostomy or ileostomies).af. | 24347 |
| 2 | (randomized or clinical trial or clinical trials or RCT or RCTs).af. | 3854171 |
| 3 | 1 AND 2 | 2416 |

Table S2. Baseline characteristics of included RCTs.

| Study | Registration ID | Inclusion criteria | Exclusion criteria | Index operation | Groups | Sample size | BMI  (kg/m^2^) | Rectal cancer | Tumor location | Neoadjuvant |
| --- | --- | --- | --- | --- | --- | --- | --- | --- | --- | --- |
| Alves 2008 | NCT00428636 | All patients over 18 years old with benign or malignant disease requiring elective rectal resection with a low anastomosis (7 cm or less above the anal verge) | Contraindications to early closure of the temporary loop ileostomy, such as signs of active infection or organ failure in the postoperative period, or radiological signs of anastomotic leakage evident at a water-soluble contrast examination through the temporary loop ileostomy performed 7 days after surgery | Rectal resection with low colorectal, coloanal or ileoanal anastomosis | Early | 95 | 23±3 ^^^ | 61 | NR | 55 |
|  |  |  |  |  | Intermediate | 91 | 24±4 ^^^ | 60 | NR | 51 |
| Lasithiotakis 2016 | NR | All consecutive patients having a defunctioning ileostomy during a low rectal or anal anastomosis and with satisfactory gastrografin enema on postoperative day 6 | Patients on steroids, at high cardiorespiratory risk, and those experiencing any postoperative complication (primary operation) or a radiological leak | Rectal resection with low rectal or anal anastomosis | Early | 16 | NR | 7 | NR | 4 |
|  |  |  |  |  | Intermediate | 10 | NR | 5 | NR | 2 |
| Danielson 2017  Park 2018  Keane 2019 | NCT01287637 | Patients were > 18 years old without any clinical sign of postoperative complications after the index operation (infections or clinical signs of leakage) | Patient with a leak of contrast outside the rectum at the CT scan with a water-soluble contrast medium. Patients with diabetes, patients being treated with steroids, patients with linguistic difficulties, and patients with expected low compliance | Total mesorectal excision for rectal cancer | Early | 55 | 24(17-32) ^#^ | 55 | Middle third 27  Upper third 27 | 16 |
|  |  |  |  |  | Late | 57 | 23(19-35) ^#^ | 57 | Middle third 24  Upper third 33 | 16 |
| Kłęk 2018 | NCT02997267 | Patients were ≥ 18 years old who had undergone anterior resection of the rectum with protective loop ileostomy for rectal adenocarcinoma | Colorectal malignancy other than rectal adenocarcinoma, protective loop ileostomy performed for other indications (part of treatment for postoperative complication) | Low anterior resection of the rectum | Early | 29 | NR | 29 | NR | 29 |
|  |  |  |  |  | Late | 29 | NR | 29 | NR | 29 |
| Gallyamov 2019 | NR | Total or partial mesorectal excision for rectal cancer with formation of a defunctioning ileostomy | Radiological or endoscopic signs of anastomotic insufficiency, diabetes, steroid treatment, expected low compliance | Total or partial mesorectal excision | Early | 31 | 24(17-33) ^#^ | 31 | Middle third 15  Upper third 15 | 9 |
|  |  |  |  |  | Late | 34 | 23(18-35) ^#^ | 34 | Middle third 14  Upper third 20 | 9 |
| Bausys 2019  Dulskas 2021 | NCT03796702 | Patients over 18 years old with rectal cancer were screened and included in the study after the elective rectal resection with a temporary loop ileostomy | Contraindications to the closure of temporary ileostomy, such as radiological/endoscopic or clinical signs of colorectal anastomosis insufficiency, also general contraindications for surgery such as signs of active infection or organ failure, which would contraindicate ileostomy closure 30 days after creation | Elective rectal resection with colorectal anastomosis (lower than 6 cm from the anal verge) | Early | 43 | NR | 43 | Lower third 7  Middle third 27  Upper third 9 | 20 |
|  |  |  |  |  | Intermediate | 38 | NR | 38 | Lower third 8  Middle third 23  Upper third 7 | 19 |
| Elsner 2021 | NCT02609451 | Patients were > 18 years old; patient with rectal cancer who underwent low anterior resection; anastomosis ≤ 5 cm from the anal verge with ileostomy | Pregnancy, allergy to contrast agent, limited contractual capability, and abdominopelvic or severe non-surgical complications | Low anterior resection with anastomosis ≤5 cm from the anal verge | Early | 37 | 25(18-33) ^#^ | 37 | NR | 13 |
|  |  |  |  |  | Intermediate | 34 | 25(17-36) ^#^ | 34 | NR | 12 |
| Ellebæk 2023 | NCT01865071 | Patients were ≥18 years old, have a histologically proven adenocarcinoma of the rectum at or below 15 cm from the anal verge, and scheduled for low anterior resection with curative intention | NR | Low anterior resection with curative intention | Intermediate | 77 | 26±4 ^^^ | 77 | 8.93±2.22 ^^^ | 21 |
|  |  |  |  |  | Late | 69 | 27±6 ^^^ | 69 | 8.97±2.49 ^^^ | 16 |
| Ahmadi-Amoli 2023 | IRCT20201113049373N1 | Patients with stage two or three nonmetastatic rectal adenocarcinomas); patients receiving neoadjuvant radiotherapy; patients undergoing low anterior resection and protective loop ileostomy | Patient with a history of inflammatory bowel disease, contraindication to adjuvant chemotherapy, uncompleted chemo/radiotherapy, advanced disease stage four under neoadjuvant chemo/radiotherapy, or immunocompromise | Low anterior resection of the rectum | Intermediate | 50 | 23±1 ^^^ | 50 | Lower third 15  Middle third 30  Upper third 5 | 50 |
|  |  |  |  |  | Late | 54 | 24±1 ^^^ | 54 | Lower third 24  Middle third 26  Upper third 4 | 54 |
| Vogel 2023 | NCT03587519 | Patients between the age of 18 and 65 years with a diagnosis of ulcerative colitis who underwent nonemergent/urgent 2- or 3-stage proctocolectomy with ileac pouch-anal anastomosis | Patients who use prednisone ≥20 mg/d within 4 weeks of ileac pouch-anal anastomosis surgery, body mass index ≥40 kg/m2, hemoglobin <8 g/ dL, serum albumin <2.5 g/dL, sepsis, or organ failure (eg, renal insufficiency with serum creatinine >2 mg/dL) at the time of study enrollment | Proctocolectomy with ileac pouch-anal anastomosis | Early | 10 | 22(19-34) ^#^ | 0 | NR | NR |
|  |  |  |  |  | Intermediate | 12 | 22(18-37) ^#^ | 0 | NR | NR |

^#^ median (range), ^^^ mean±SD, NR not report.

Table S3. Evaluation of methodological qualities of included randomized controlled trials.

| Items/author | Alves 2008 | Lasithiotakis 2016 | Danielson 2017 | Kłęk 2018 | Gallyamov 2019 | Bausys 2019 | Elsner 2021 | Ellebæk 2023 | Ahmadi-Amoli 2023 | Vogel 2023 |
| --- | --- | --- | --- | --- | --- | --- | --- | --- | --- | --- |
| Described as randomized | 1 | 1 | 1 | 1 | 1 | 1 | 1 | 1 | 1 | 1 |
| Appropriate randomization method described | 1 | 1 | 1 | 1 | 1 | 1 | 1 | 1 | 1 | 1 |
| Subject blinded to intervention | 0 | 0 | 0 | 0 | 0 | 0 | 0 | 0 | 0 | 0 |
| Evaluator blinded to intervention | 0 | 0 | 0 | 0 | 0 | 0 | 0 | 0 | 0 | 0 |
| Description of withdrawals and dropouts | 1 | 1 | 1 | 1 | 1 | 1 | 1 | 1 | 1 | 1 |
| Score | 3 | 3 | 3 | 3 | 3 | 3 | 3 | 3 | 3 | 3 |

Methodological qualities of included randomized controlled trials were assessed by standard Jadad score system. Total score, 5; 1-2, low quality trial; 3-5, high quality trial.

Table S4. Direct comparison results of all outcomes.

| Outcomes | Comparison | Intermediate | Late |
| --- | --- | --- | --- |
| Total postoperative complications^#^ | Early | 2.43 (0.87, 6.78) | 0.93 (0.36, 2.38) |
|  | Intermediate | — | 0.74 (0.35, 1.53) |
| Major complications^#^ | Early | 4.39 (0.85, 22.65) | 0.69 (0.19, 2.55) |
|  | Intermediate | — | 0.60 (0.23, 1.52) |
| Leak of rectal anastomosis^#^ | Early | 1.16 (0.44, 3.08) | 0.34 (0.01, 8.51) |
|  | Intermediate | — | 3.73 (0.41, 34.17) |
| Bowel obstruction/ileus^#^ | Early | 0.70 (0.22, 2.28) | 1.04 (0.18, 6.14) |
|  | Intermediate | — | 2.83 (0.55, 14.52) |
| Wound complications^#^ | Early | 3.36 (1.46, 7.74) | 1.31 (0.18, 9.55) |
|  | Intermediate | — | — |
| Postoperative hospital stay^*^ | Early | 1.00 (0.31, 1.69) | 0.27 (-0.30, 0.84) |
|  | Intermediate | — | 0.30 (0.11, 0.49) |
| Reoperation^#^ | Early | 2.91 (0.73, 11.58) | 1.07 (0.34, 3.39) |
|  | Intermediate | — | — |
| Readmission^#^ | Early | 3.44 (0.50, 23.40) | — |
|  | Intermediate | — | — |
| Blood loss^*^ | Early | 5.00 (-9.17, 19.17) | -1.93 (-6.61, 2.75) |
|  | Intermediate | — | 10.80 (-9.10, 30.69) |
| Operative time^*^ | Early | -0.79 (-13.14, 11.56) | -9.11 (-17.43, -0.79) |
|  | Intermediate | — | 2.06 (-5.48, 9.61) |
| Stoma-related complications^#^ | Early | 0.08 (0.01, 0.61) | 0.09 (0.04, 0.22) |
|  | Intermediate | — | 0.52 (0.15, 1.80) |

^#^ odds ratio with 95% confidence interval for binary outcome comparison. ^*^ weighted mean difference with 95% confidence interval for continuous outcome comparison. “—” not applicable.

Table S5. Low anterior resection syndrome.

| Study | Follow-up (months) | LARS | Early | Intermediate | Late | P value |
| --- | --- | --- | --- | --- | --- | --- |
| Ahmadi-Amoli 2023 | NR | Major | - | 10 | 17 | 0.356 |
|  |  | Minor | - | 15 | 18 |  |
|  |  | No | - | 22 | 22 |  |
| Dulskas 2021 | 30(17-97) ^#^ | Major | 6 | 8 | - | 0.858 |
|  |  | Minor | 6 | 6 | - |  |
|  |  | No | 14 | 11 | - |  |
| Keane 2019 | early 52(44-59) ^#^  late 44(35-53) ^#^ | Major | 25 | - | 29 | 1.528 |
|  |  | Minor | 7 | - | 5 |  |
|  |  | No | 10 | - | 6 |  |

^#^ median (range), NR not report, “-” not applicable.

Table S6. Gastrointestinal Quality of Life Index (GIQLI) questionnaire.

| Study | Time point | Patients | Early | Intermediate | Late | P value |
| --- | --- | --- | --- | --- | --- | --- |
| Ellebæk 2023 | Baseline | 50 vs 44 | - | 107.06±13.65 ^^^ | 105.23±12.86 ^^^ | >0.05 |
|  | 6 months after LAR | 54 vs 46 | - | 106.07±13.44 ^^^ | 101.00±14.12 ^^^ | >0.05 |
|  | 12 months after LAR | 53 vs 53 | - | 107.09±14.34 ^^^ | 105.21±10.95 ^^^ | >0.05 |
| Elsner 2021 | Preoperatively | 37 vs 34 | 109.5(39-143) ^#^ | 117.5(69-142) ^#^ | - | 0.258 |
|  | 6 weeks after LAR | 37 vs 34 | 97.0(55-130) ^#^ | 108.0(59-132) ^#^ | - | 0.139 |
|  | 4 months after LAR | 37 vs 34 | 106.0(75-143) ^#^ | 109.0(75-134) ^#^ | - | 0.904 |
| Alves 2008 | 12 months | 63 vs 70 | 111(43-141) ^#^ | 108(37-139) ^#^ | - | 0·566 |

^#^ median (range), ^^^ mean±SD, LAR low anterior resection, “-” not applicable.

Table S7. Direct comparison results of subgroup analysis.

| Subgroup analysis | Comparison | Intermediate | Late |  |
| --- | --- | --- | --- | --- |
| **Rectal cancer** | | | |  |
| Total postoperative complications | Early | 2.88 (1.30, 6.37) | 0.93 (0.36, 2.38) |  |
|  | Intermediate | - | 0.74 (0.35, 1.53) |  |
| Major complications | Early | 12.52 (1.59, 98.92) | 0.69 (0.19, 2.55) |  |
|  | Intermediate | - | 0.60 (0.23, 1.52) |  |
| **Time points of randomization (after evaluating the integrity of the anastomosis)** | | | |  |
| Total postoperative complications | Early | 2.73 (0.65, 11.54) | 0.93 (0.36, 2.38) |  |
|  | Intermediate | - | 0.50 (0.20, 1.22) |  |
| Major complications | Early | 3.26 (0.53, 20.00) | 0.69 (0.19, 2.55) |  |
|  | Intermediate | - | 0.71 (0.11, 4.42) |  |
| **After evaluating the integrity of the anastomosis in rectal cancer** | | | |  |
| Total postoperative complications | Early | 4.52 (1.17, 17.50) | 0.93 (0.36, 2.38) |  |
|  | Intermediate | - | 0.50 (0.20, 1.22) |  |
| Major complications | Early | 11.00 (0.59, 205.86) | 0.69 (0.19, 2.55) |  |
|  | Intermediate | - | 0.71 (0.11, 4.42) |  |
| **Very early vs intermediate vs late closure** | | | | |
| Total postoperative complications | Very early | 2.08 (0.66, 6.55) | 1.06 (0.33, 3.42) | |
|  | Intermediate | - | 0.74 (0.35, 1.53) | |
| Major complications | Very early | 3.73 (0.53, 26.41) | 0.62 (0.14, 2.73) | |
|  | Intermediate | - | 0.60 (0.23, 1.52) | |
| **Very early vs intermediate vs late closure in rectal cancer** | | | | |
| Total postoperative complications | Very early | 2.27 (0.85, 6.06) | 1.06 (0.33, 3.42) | |
|  | Intermediate | - | 0.74 (0.35, 1.53) | |
| Major complications | Very early | 14.24 (0.77, 263.13) | 0.62 (0.14, 2.73) | |
|  | Intermediate | - | 0.60 (0.23, 1.52) | |
| **Very early vs intermediate vs late closure after evaluating the integrity of the anastomosis** | | | | |
| Total postoperative complications | Very early | 2.38 (0.37, 15.38) | 1.06 (0.33, 3.42) | |
|  | Intermediate | - | 0.50 (0.20, 1.22) | |
| Major complications | Very early | 2.27 (0.26, 19.68) | 0.62 (0.14, 2.73) | |
|  | Intermediate | - | 0.71 (0.11, 4.42) | |
| **Very early vs intermediate vs late closure after evaluating the integrity of the anastomosis in rectal cancer** | | | | |
| Total postoperative complications | Very early | - | 1.06 (0.33, 3.42) | |
|  | Intermediate | - | 0.50 (0.20, 1.22) | |
| Major complications | Very early | - | 0.62 (0.14, 2.73) | |
|  | Intermediate | - | 0.71 (0.11, 4.42) | |
| **Rectal cancer subgroup (LAR/TME cohort)** | | | | |
| Total postoperative complications | Early | - | 0.89 (0.34, 2.34) | |
|  | Intermediate | - | 0.78 (0.46, 1.35) | |
| Major complications | Early | - | 0.62 (0.15, 2.55) | |
|  | Intermediate | - | 0.62(0.27, 1.47) | |

Figure S1. Risk of bias summary.

Figure S2. Comparison network (a), forest plot results and GRADE (b), convergence diagram (c), trajectory density diagram (d), rank probabilities(e) and consistency and inconsistency models (node-splitting method) (f) of total postoperative complications. A early group, B intermediate group, C late group. SUCRA surface under the cumulative ranking curve.

Figure S3. Comparison network (a), forest plot results and GRADE (b), convergence diagram (c), trajectory density diagram (d), rank probabilities(e) and consistency and inconsistency models (node-splitting method) (f) of major complications. A early group, B intermediate group, C late group. SUCRA surface under the cumulative ranking curve.

Figure S4. Comparison network (a), forest plot results and GRADE (b), convergence diagram (c), trajectory density diagram (d), rank probabilities(e) and consistency and inconsistency models (node-splitting method) (f) of leak of rectal anastomosis. A early group, B intermediate group, C late group. SUCRA surface under the cumulative ranking curve.

Figure S5. Comparison network (a), forest plot results and GRADE (b), convergence diagram (c), trajectory density diagram (d), rank probabilities(e) and consistency and inconsistency models (node-splitting method) (f) of bowel obstruction/ileus. A early group, B intermediate group, C late group. SUCRA surface under the cumulative ranking curve.

Figure S6. Comparison network (a), forest plot results and GRADE (b), convergence diagram (c), trajectory density diagram (d) and rank probabilities(e) of wound complications. A early group, B intermediate group, C late group. SUCRA surface under the cumulative ranking curve.

Figure S7. Comparison network (a), forest plot results and GRADE (b), convergence diagram (c), trajectory density diagram (d), rank probabilities(e) and consistency and inconsistency models (node-splitting method) (f) of postoperative hospital stay. A early group, B intermediate group, C late group. SUCRA surface under the cumulative ranking curve.

Figure S8. Comparison network (a), forest plot results and GRADE (b), convergence diagram (c), trajectory density diagram (d) and rank probabilities(e) of reoperation. A early group, B intermediate group, C late group. SUCRA surface under the cumulative ranking curve.

Figure S9. Comparison network (a), forest plot results and GRADE (b), convergence diagram (c), trajectory density diagram (d) and rank probabilities(e) of readmission. A early group, B intermediate group, C late group. SUCRA surface under the cumulative ranking curve.

Figure S10. Comparison network (a), forest plot results and GRADE (b), convergence diagram (c), trajectory density diagram (d), rank probabilities(e) and consistency and inconsistency models (node-splitting method) (f) of operative time. A early group, B intermediate group, C late group. SUCRA surface under the cumulative ranking curve.

Figure S11. Comparison network (a), forest plot results and GRADE (b), convergence diagram (c), trajectory density diagram (d), rank probabilities(e) and consistency and inconsistency models (node-splitting method) (f) of blood loss. A early group, B intermediate group, C late group. SUCRA surface under the cumulative ranking curve.

Figure S12. Comparison network (a), forest plot results and GRADE (b), convergence diagram (c), trajectory density diagram (d), rank probabilities(e) and consistency and inconsistency models (node-splitting method) (f) of stoma-related complications. A early group, B intermediate group, C late group. SUCRA surface under the cumulative ranking curve.

Figure S13. Comparison network (a), forest plot results and GRADE (b), convergence diagram (c), trajectory density diagram (d), rank probabilities(e) and consistency and inconsistency models (node-splitting method) (f) of total postoperative complications for rectal cancer. A early group, B intermediate group, C late group. SUCRA surface under the cumulative ranking curve.

Figure S14. Comparison network (a), forest plot results and GRADE (b), convergence diagram (c), trajectory density diagram (d), rank probabilities(e) and consistency and inconsistency models (node-splitting method) (f) of major complications for rectal cancer. A early group, B intermediate group, C late group. SUCRA surface under the cumulative ranking curve.

Figure S15. Comparison network (a), forest plot results and GRADE (b), convergence diagram (c), trajectory density diagram (d), rank probabilities(e) and consistency and inconsistency models (node-splitting method) (f) of total postoperative complications for time points of randomization (after evaluating the integrity of the anastomosis). A early group, B intermediate group, C late group. SUCRA surface under the cumulative ranking curve.

Figure S16. Comparison network (a), forest plot results and GRADE (b), convergence diagram (c), trajectory density diagram (d), rank probabilities(e) and consistency and inconsistency models (node-splitting method) (f) of major complications for time points of randomization (after evaluating the integrity of the anastomosis). A early group, B intermediate group, C late group. SUCRA surface under the cumulative ranking curve.

Figure S17. Comparison network (a), forest plot results and GRADE (b), convergence diagram (c), trajectory density diagram (d), rank probabilities(e) and consistency and inconsistency models (node-splitting method) (f) of total postoperative complications after evaluating the integrity of the anastomosis in rectal cancer. A early group, B intermediate group, C late group. SUCRA surface under the cumulative ranking curve.

Figure S18. Comparison network (a), forest plot results and GRADE (b), convergence diagram (c), trajectory density diagram (d), rank probabilities(e) and consistency and inconsistency models (node-splitting method) (f) of major complications after evaluating the integrity of the anastomosis in rectal cancer. A early group, B intermediate group, C late group. SUCRA surface under the cumulative ranking curve.

Figure S19. Comparison network (a), forest plot results and GRADE (b), convergence diagram (c), trajectory density diagram (d), rank probabilities(e) and consistency and inconsistency models (node-splitting method) (f) of total postoperative complications for very early (stoma closure within 15 days) vs intermediate vs late closure. A very early group (stoma closure within 15 days), B intermediate group, C late group. SUCRA surface under the cumulative ranking curve.

Figure S20. Comparison network (a), forest plot results and GRADE (b), convergence diagram (c), trajectory density diagram (d), rank probabilities(e) and consistency and inconsistency models (node-splitting method) (f) of major complications for very early (stoma clousure within 15 days) vs intermediate vs late closure. A very early group (stoma clousure within 15 days), B intermediate group, C late group. SUCRA surface under the cumulative ranking curve.

Figure S21. Comparison network (a), forest plot results and GRADE (b), convergence diagram (c), trajectory density diagram (d), rank probabilities(e) and consistency and inconsistency models (node-splitting method) (f) of total postoperative complications for very early (stoma clousure within 15 days) vs intermediate vs late closure in rectal cancer. A very early group (stoma clousure within 15 days), B intermediate group, C late group. SUCRA surface under the cumulative ranking curve.

Figure S22. Comparison network (a), forest plot results and GRADE (b), convergence diagram (c), trajectory density diagram (d), rank probabilities(e) and consistency and inconsisten cy models (node-splitting method) (f) of major complications for very early (stoma clousure within 15 days) vs intermediate vs late closure in rectal cancer. A very early group (stoma clousure within 15 days), B intermediate group, C late group. SUCRA surface under the cumulative ranking curve.

Figure S23. Comparison network (a), forest plot results and GRADE (b), convergence diagram (c), trajectory density diagram (d), rank probabilities(e) and consistency and inconsistency models (node-splitting method) (f) of total postoperative complications for very early (stoma clousure within 15 days) vs intermediate vs late closure after evaluating the integrity of the anastomosis. A very early group (stoma clousure within 15 days), B intermediate group, C late group. SUCRA surface under the cumulative ranking curve.

Figure S24. Comparison network (a), forest plot results and GRADE (b), convergence diagram (c), trajectory density diagram (d), rank probabilities(e) and consistency and inconsisten cy models (node-splitting method) (f) of major complications for very early (stoma clousure within 15 days) vs intermediate vs late closure after evaluating the integrity of the anastomosis. A very early group (stoma clousure within 15 days), B intermediate group, C late group. SUCRA surface under the cumulative ranking curve.

Figure S25. Comparison network (a), forest plot results and GRADE (b), convergence diagram (c), trajectory density diagram (d) and rank probabilities(e) of total postoperative complications for very early (stoma clousure within 15 days) vs intermediate vs late closure after evaluating the integrity of the anastomosis in rectal cancer. A very early group (stoma clousure within 15 days), B intermediate group, C late group. SUCRA surface under the cumulative ranking curve.

Figure S26. Comparison network (a), forest plot results and GRADE (b), convergence diagram (c), trajectory density diagram (d) and rank probabilities(e) of major complications for very early (stoma clousure within 15 days) vs intermediate vs late closure after evaluating the integrity of the anastomosis in rectal cancer. A very early group (stoma clousure within 15 days), B intermediate group, C late group. SUCRA surface under the cumulative ranking curve.

Figure S27. Comparison network (a), forest plot results (b), convergence diagram (c), trajectory density diagram (d), rank probabilities(e) and consistency and inconsistency models (node-splitting method) (f) of total postoperative complications in the rectal cancer subgroup (LAR/TME cohort). A early group, B intermediate group, C late group. SUCRA surface under the cumulative ranking curve. LAR low anterior resection, TME, total mesorectal excision.

Figure S28. Comparison network (a), forest plot results (b), convergence diagram (c), trajectory density diagram (d) and rank probabilities(e) of major postoperative complications in the rectal cancer subgroup (LAR/TME cohort). A early group, B intermediate group, C late group. SUCRA surface under the cumulative ranking curve. LAR low anterior resection, TME, total mesorectal excision.

Figure S29. Adjusted funnel plot for total postoperative (a) and major complications.
